# Associations Between Eating Windows and Health Outcomes in Children and Adolescents from the ALSPAC Cohort

**DOI:** 10.3390/nu17172856

**Published:** 2025-09-03

**Authors:** Jill Townley, Sam Leary, Julian Hamilton-Shield, Melanie de Lange, Elanor C. Hinton, Kate Northstone

**Affiliations:** 1 Bristol Dental School, University of Bristol, Dorothy Hodgkin Building, Whitson Street, Bristol BS1 3NY, UK; 2 NIHR Bristol Biomedical Research Centre, Diet and Physical Activity Theme, Faculty of Health Sciences, University of Bristol, Oakfield House, Oakfield Grove, Bristol BS8 2BN, UK; j.p.h.shield@bristol.ac.uk (J.H.-S.); elanor.hinton@bristol.ac.uk (E.C.H.); 3 Bristol Dental School, University of Bristol, 1 Trinity Walk, Bristol BS2 0PT, UK; s.d.leary@bristol.ac.uk; 4 MRC Integrative Epidemiology Unit, University of Bristol, Oakfield House, Oakfield Grove, Bristol BS8 2BN, UK; melanie.delange@bristol.ac.uk; 5 Population Health Sciences, University of Bristol, Learning & Research Building, Level 1, Southmead Hospital, Bristol BS10 5NB, UK; kate.northstone@bristol.ac.uk

**Keywords:** eating windows, eating duration, time-limited eating, children, adolescents, chrono-nutrition, body composition, metabolic health, ALSPAC

## Abstract

Background: Time-limited eating (TLE) improves body weight and metabolic health in adults; however, little is known about effects in younger populations (YP). TLE in adolescents can reduce calorie consumption, but studies have not demonstrated superior weight loss compared to other dietary practices. Minimal research exists into associations between eating window (EW) in YP and health outcomes. Methods: Three-day diet diaries (ages 7, 13 years) collected in the Avon Longitudinal Study of Parents and Children (ALSPAC) were used to calculate EW. Height, weight, body composition, blood pressure, and fasting bloods were recorded during clinic visits at ages 7, 13, and 24. Linear or logistic regression models were used to analyse cross-sectional and longitudinal associations, accounting for potential confounders. Results: Mean EW was 10.9 h (standard deviation 1.1) and 11.1 h (1.8) at ages 7 and 13, respectively. At age 7 (N = 4799), a longer EW was positively associated with body mass index z-score (BMIz) (beta coefficient (β) 0.04 (95% confidence interval 0.01, 0.07) *p* = 0.01), whilst at age 13 (N = 4712) a longer EW showed inverse associations with BMIz (β −0.026 (−0.046, −0.006) *p* = 0.01), waist to height ratio (WtHR) (β −0.001 (−0.002, −0.000) *p* = 0.005), waist circumference (WC) (cm) (β −0.211 (−0.370, −0.053) *p* = 0.009), diastolic blood pressure (DBP) (mmHg) (β −0.132 (−0.254, −0.009) *p* = 0.04), and fat mass (FM) (%) (β −0.447 (−0.607, −0.286) *p* < 0.001). Longitudinally, a longer EW at age 13 (N = 2534) was inversely associated with FM (%) at age 24 (β −0.307 (−0.487, −0.127) *p* < 0.001). Conclusions: A longer EW in adolescence was associated cross-sectionally with lower BMIz, WtHR, WC, DBP, and FM and longitudinally with lower FM at age 24, albeit with small effect sizes.

## 1. Introduction

Chrono-nutrition is a rapidly expanding field of research investigating the relationships between timing of eating, circadian rhythms, and metabolic health [1]. Circadian rhythms are the approximately 24 h oscillations in bodily responses in relation to the Earth’s 24 h rotation and include sleep cycles and hormone fluctuations. Whilst circadian rhythms are controlled by a ‘master clock’ located in the suprachiasmatic nucleus within the hypothalamus, environmental cues, known as ‘zeitgebers’, such as daylight or eating times, can affect circadian rhythms. Many bodily functions are known to be governed by circadian rhythms, with diurnal fluctuations seen in hormones including insulin, melatonin, and cortisol [2]. Insulin sensitivity is known to be greater earlier in the day; identical meals result in higher post-prandial blood glucose levels when consumed in the evening compared to the morning [3]. Additionally, the energy used to digest, absorb, and store nutrients from meals, known as diet-induced thermogenesis (DIT), is shown to be higher for meals consumed in the morning [4]. This, alongside reduced post-prandial glucose, suggests metabolic benefits to eating earlier.

Time-limited eating (TLE) is a dietary pattern where the focus is not on *what* is eaten but rather *when* food is eaten. Consequently, there are no restrictions on calorie intake or other elements of meal composition. Instead, food and beverage intake is restricted to a specific window within any 24 h period, whereby all calorific intake is consumed within this time frame. Typically, this would be between 8 and 12 h, thus giving a 12–16 h fasting period. Whilst TLE can inadvertently result in calorie reduction [5], the benefits are thought to exist independently of this [6]. Additionally, fasting periods in excess of 12 h are thought to lead to a metabolic switch; glucose reserves are depleted, so the body switches to using fat stores as fuel, leading to reduced fat mass whilst preserving lean mass [7]. TLE is thought to work alongside our innate circadian rhythms, with more favourable bodily responses to food and the benefits of longer fasting periods thought to have a range of metabolic health benefits.

Adults have been shown to have a long eating window (EW), often exceeding 14 h [5,8,9]; however, considerable variation is seen in this [8]. Within adult populations, TLE has been shown to reduce body weight [5], glycated haemoglobin (HbA1c) and blood pressure [5,9], total cholesterol (TC), and low-density lipoprotein cholesterol (LDL-C) levels [5] and increase high-density lipoprotein cholesterol (HDL-C) levels [10].

Whilst there is less literature on the EW of children and adolescents, a recent systematic review showed an average EW exceeding 11 h; however, considerable variation was seen between individual studies [11]. Limited research exists on the health benefits of TLE within child and adolescent populations; pilot studies have shown TLE interventions to be associated with a reduction in calorie consumption in adolescents [12,13], consistent with the adult literature [14,15], but with no apparent effect on weight or blood glucose levels compared to controls.

The transition from childhood to adolescence is known to involve a shift in chronotype, with a change from a morning chronotype, waking early and going to bed early, to a later evening chronotype, where adolescents wake later and go to bed later [16]. Less is known about how this chronotype shift influences eating patterns and whether this has any impact on weight status or metabolic health. There remains a need for further research into EW in childhood and adolescence and whether there are associations between EW and anthropometric and metabolic outcomes in childhood, adolescence, and into early adulthood. Therefore, this study aims to quantify the length of daily EW at ages 7 and 13 and assess both cross-sectional and longitudinal associations between daily EW in childhood (age 7) and adolescence (age 13) with measures of body composition and metabolic markers in childhood, adolescence, and early adulthood (age 24).

This study will address three research questions: 1. What is the average eating window of children (aged 7 years) and adolescents (aged 13 years)? 2. Are eating windows in childhood and adolescence associated with changes in body composition and metabolic health? 3. Are eating windows in childhood and adolescence associated with changes in body composition and metabolic health in early adulthood (aged 24 years)? We hypothesised that a longer EW at ages 7 and 13 years will be associated with increased body mass index z-score (BMIz), waist to height ratio (WtHR), waist circumference (WC), fat mass (FM), and normal weight obesity (NWO) in childhood and adolescence and with increased BMI, WtHR, WC, FM, NWO, systolic blood pressure (SBP), diastolic blood pressure (DBP), TC, LDL-C, triglycerides, and fasting glucose (FG) and reduced HDL-C in early adulthood.

## 2. Materials and Methods

### 2.1. Sample

This study used data from participants enrolled in the Avon Longitudinal Study of Parents and Children (ALSPAC), a birth cohort study where pregnant women with expected delivery dates between 1 April 1991 and 31 December 1992 and residing in Avon, UK, were eligible to participate. Of 20,248 eligible pregnancies, 14,541 pregnancies were initially enrolled, resulting in 13,988 children alive at 1 year of age. Three further phases of recruitment resulted in enrolment of an additional 913 children, giving a sample size of 14,901 children alive at 1 year of age [17,18,19]. Full details are available at www.bristol.ac.uk/alspac/ (accessed 3 February 2025). Study data were collected and managed using REDCap electronic data capture tools hosted at the University of Bristol [20]. REDCap (Research Electronic Data Capture) is a secure, web-based software platform designed to support data capture for research studies. The study website contains details of all of the data available through a fully searchable data dictionary and a variable search tool (https://www.bristol.ac.uk/alspac/researchers/our-data/) (accessed 24 March 2025).

Ethical approval for the study was obtained from the ALSPAC Ethics and Law Committee and the Local Research Ethics Committees. Consent for biological samples was collected in accordance with the Human Tissue Act (2004). Informed consent for the use of data collected via questionnaires and clinics was obtained from participants following the recommendations of the ALSPAC Ethics and Law Committee at the time (https://www.bristol.ac.uk/alspac/researchers/research-ethics/) (accessed 1 May 2025).

### 2.2. Dietary Assessment

Dietary intake was assessed via a 3-day dietary diary at ages 7 and 13 completed by parents at age 7 and by adolescents at age 13, with parental assistance as needed. These were completed over three days (one weekend day and two weekdays) just prior to a face-to-face assessment. Full details have been described previously [21]. Briefly, children and/or their parents recorded all food and beverage intake using standard household measures (e.g., number of spoonfuls or large bowlful). There were sections to record the exact time of intake and a section to record anything not eaten. Diaries were checked by a nutritionist at the face-to-face visits to review inconsistencies and clarify portion sizes. Dietary records were coded using the DIDO (Data In, Diet Out) programme, developed by the Human Nutrition Research Unit, Cambridge [22], whilst nutritional analysis was performed using McCance and Widdowson’s British food composition tables [23].

### 2.3. Exposure Variable

#### Eating Window

EW was operationalised as the time from the first to the last recorded calorie intake of ≥10 kcal within any individual 24 h period. Eating time was converted to a decimal, with hours remaining the same but minutes divided by 60, e.g., 08:30 a.m. = 8.50. The mean EW across all recorded days was calculated using decimalised times. There is inconsistency in the existing literature regarding minimum calorie consumption to define an eating occasion, with previous research using >0 kcal [24], ≥10 kcal [25], or ≥50 kcal [26] as a minimum calorie intake. In this study, ≥10 kcal was used to enable consistency with previous research in child and adolescent populations.

### 2.4. Outcome Variables

Participants attended face-to-face assessments at 7, 13, and 24 years of age, where measurements and blood samples were collected using standardised procedures.

#### 2.4.1. Body Composition

Height was measured to the nearest mm using the Harpenden Stadiometer (Holtain Ltd., Crymych, UK). Weight was measured to the nearest 0.1 kg using the Tanita Body Fat Analyser (Tanita UK Ltd., Middlesex, UK), with the child wearing underclothes. BMI was calculated as weight (kg)/height (m)^2^. At ages 7 and 13, BMI z-scores were calculated using 1990 British Growth Reference data [27]. WC was measured to the nearest mm, at the narrowest point between the iliac crests and the bottom ribs, using Harpenden anthropometric tape. WtHR was calculated as WC (cm)/height (cm). FM was measured, in grams (g), via dual-energy X-ray absorptiometry (DXA) at ages 13 and 24, and percentage body fat was calculated from DXA measurements as total fat mass (g)/total body mass (bone (g) + lean (g) + fat (g)) × 100.

NWO is defined as excessive body fat with a normal BMI [28] and is associated with increased cardiometabolic risk [29,30]. The diagnostic criteria for NWO in children and young people (CYP) vary within the literature [31], with different cut-off values of both excess body fat and BMI depending on country-specific reference values. Due to the use of British CYP in this paper, we defined NWO using British reference values, BMIz < 1.34 standard deviations (SD) at age 13 and BMI < 25 kg/m^2^ at age 24 [32], and body fat percentages ≥ 22.0/29.4% at age 13 and ≥20.1/30.8% at age 24 for males/females, respectively [33].

#### 2.4.2. Blood Pressure

At ages 7 and 13, blood pressure was measured using a Dinamap 9301 Vital Signs monitor (Morton Medical, London, UK). Two readings were taken, and mean systolic and diastolic pressure was calculated. At age 24, blood pressure was measured using an Omron M6 monitor (Omron Electronic Components Europe BV). Up to three readings were taken, and the mean systolic and diastolic pressure was calculated.

#### 2.4.3. Blood Samples

Blood samples for fasting glucose and lipid profiles (TC, triglycerides, HDL-C, and LDL-C) were collected at age 24 following an overnight, or minimum 8 h, fast. Fasting glucose was measured using the hexokinase method and TC, triglycerides, and HDL-C were measured using enzymatic colorimetric tests, whilst LDL-C was calculated using the Friedewald equation (full details available at https://www.bristol.ac.uk/alspac/researchers/our-data/) (accessed 24 March 2025).

### 2.5. Confounding Variables

Data on potential confounders were collected via medical records, questionnaires, and face-to-face assessments throughout the study. Child age, in months, was derived at face-to-face visits using date of attendance and date of birth. Child sex was recorded from birth records. Pubertal status was assessed using age of peak height velocity (aPHV), an objective measure derived using Superimposition by Translation and Rotation (SITAR) [34]. Maternal pre-pregnancy BMI, maternal date of birth (from which age at delivery was derived), maternal education, and maternal and paternal occupation were collected using a questionnaire during pregnancy. Maternal education was categorised as none/CSE, vocational (job-specific qualifications), O-level (academic qualifications at age 16), A-level (academic qualifications at age 18), or degree (university qualification). Social class was based on National Statistics Socioeconomic Classification (NS-SEC) occupation categories (I (highest), II, III (non-manual), III (manual), IV, V (lowest)), and household social class was derived using the highest of maternal or paternal NS-SEC categories. Average daily energy intake (kcal) was calculated from the diet diaries. Diet quality was assessed using the Children’s relative Mediterranean-style diet (Cr-Med) score [35] and the Children’s Dietary Inflammatory (cDIS) score [36]. Cr-Med determines whether dietary consumption aligns with a Mediterranean-style diet; higher scores represent closer alignment. cDIS reflects the inflammatory potential of dietary consumption, with higher scores being more pro-inflammatory. Low Cr-Med and high cDIS scores are associated with increased cardiometabolic risk in this cohort [35,36].

### 2.6. Statistical Analysis

Stata (version 18) was used for all analyses. Distribution of data was assessed through visual inspection of histograms. Continuous variables were described using means and SD for normally distributed continuous variables, medians and interquartile range (IQR) for non-normally distributed variables, and numbers and percentages (%) for categorical variables. Multivariable linear and logistic regression models were fitted to assess the cross-sectional associations between exposure (mean EW) at each timepoint (7 or 13) with each outcome variable (BMIz, WtHR, WC, FM, NWO, SBP, DBP). Additionally, multivariable linear and logistic regression models were used to assess longitudinal associations between exposure variables (mean EW at ages 7 or 13) and outcomes at age 24 (BMI, WtHR, WC, FM, NWO, SBP, DBP, TC, HDL-C, LDL-C, triglycerides, and FG).

Cross-sectional (age 7) and longitudinal (exposure at age 7 or 13 with outcomes at age 24) models were adjusted as follows:

- Model 1—Mean EW and each outcome variable, adjusted for age and sex.
- Model 2—Additionally adjusted for maternal and household factors: mother’s age, mother’s BMI, mother’s education level, and household social class.
- Model 3—Additionally adjusted for diet quality scores and energy intake.

Cross-sectional analysis at age 13 was additionally adjusted for pubertal status:

- Model 1—Mean EW and each outcome variable, adjusted for age and sex.
- Model 2—Additionally adjusted for pubertal status.
- Model 3—Additionally adjusted for maternal and household factors: mother’s age, mother’s BMI, mother’s education level, and household social class.
- Model 4—Additionally adjusted for diet quality scores and energy intake.

**Figure 1:**
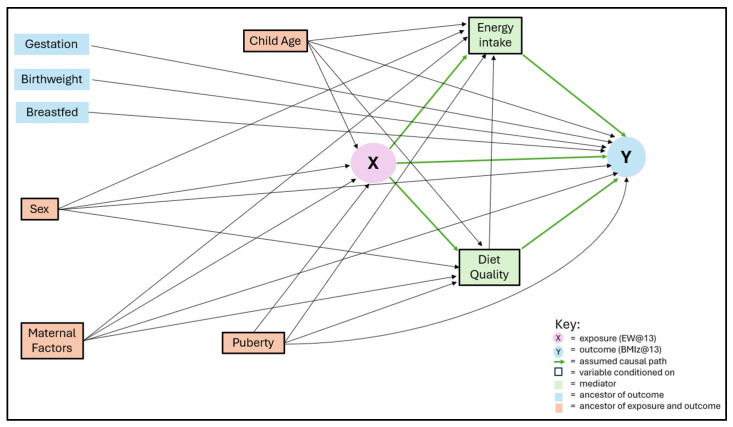
Directed acyclic graph depicting assumed causal relationships between eating window and body mass index z-score at age 13.

Directed acyclic graphs (DAGs) were drawn to identify assumed causal relationships between all exposure/outcome variable combinations (Figure 1). Energy intake and diet quality were planned to be included in the models as confounders; however, the DAGs suggested these were mediating variables. These were included in the final model, with the caveat that this model may be overfitted.

Multiple testing increases the likelihood of small *p*-values by chance. Conclusions drawn from these analyses were based on effect sizes and confidence intervals, and *p*-values were interpreted in terms of strength of the evidence against the null hypothesis [37].

### 2.7. Sensitivity Analysis

Model 1 was repeated including only participants with complete confounder data, thus checking that changes in effect sizes in models 2, 3, or 4 could be attributed to confounding rather than as a consequence of missing data.

To assess the robustness of findings, models 2 and 3 (at age 7 and longitudinal analyses) and models 3 and 4 (at age 13) were re-run using the mean EW derived from complete (3 day) dietary data and additionally re-run using the mean EW derived from diaries including both a week and a weekend day.

EW@13: eating window at age 13 years old; BMIz@13: body mass index z-score at 13 years old; maternal factors: pre-pregnancy maternal age, body mass index, education level, and household social class.

## 3. Results

### 3.1. Sample Characteristics

**Figure 2:**
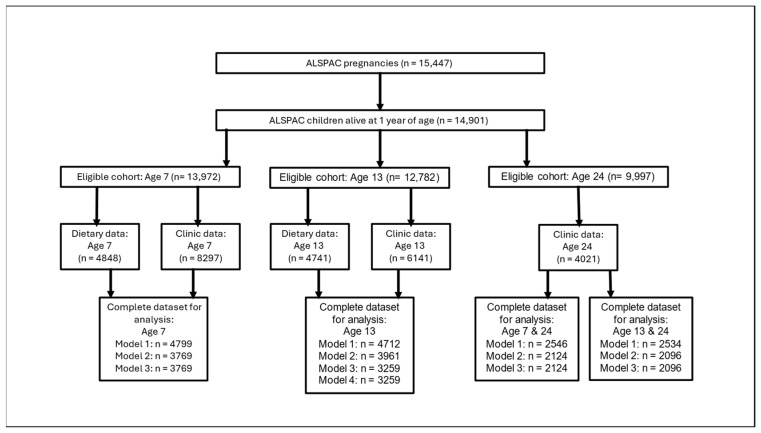
Number of participants available for analysis at each timepoint.

From the original cohort of 14,901 children alive at one year of age, 8297, 6141, and 4021 attended the face-to-face assessments at ages 7, 13, and 24, respectively. Of these, 4848 and 4791 had dietary data at ages 7 and 13, respectively (Figure 2). The mean age of attendance at the 7-year assessment was 7.5 (SD 0.3) years, 54% were female, and 96% were white (Table 1). The mean age of attendance at the 13-year assessment was 13.8 (SD 0.2) years, and 52% were female and 96% were white, whilst at the age 24 assessment mean age of attendance was 24.0 (SD 0.8) years, 63% were female, and 96% were white.

CYP who attended any assessments were more likely to have mothers who were older, better educated, and of a higher household social class than CYP who did not attend clinics (Table S1).

### 3.2. Eating Windows

#### 3.2.1. Age 7

Mean EW ranged from 4.5 to 16.3 h across individual participants, with a mean EW of 10.9 h (SD 1.1) across the whole sample (N = 4848) (Table 2). Weekday EW was 11.1 h (SD 1.2), whilst weekend EW was 10.5 h (SD 1.4). First eating times were later on weekends (8.60 AM (SD 0.9)) than weekdays (7.92 AM (SD 0.6)), whilst there was little difference in last eating time (19.03 PM (SD 1.1) vs. 19.08 PM (SD 1.2), weekdays and weekends, respectively). The mean number of meals and snacks consumed each day was 4.6 (SD 0.7).

#### 3.2.2. Age 13

Mean EW ranged from 3.5 to 16.5 h across individual participants, with a mean EW of 11.1 h (SD 1.8) across the whole sample (N = 4741). Weekday EW was 11.5 h (SD 1.9), whilst weekend EW was 10.3 (SD 2.0). First eating times were later on weekends (9.53 AM (SD 1.3)) than weekdays (8.34 AM (SD 1.4)), with minimal differences in last eating times (19.79 PM (SD 1.5) vs. 19.85 PM (SD 1.6), weekdays and weekends, respectively). The mean number of meals and snacks consumed each day was 4.5 (SD 0.9).

Datasets at age 24 consist of dietary data and dietary confounders at ages 7 or 13 and clinic variables at age 24.

### 3.3. Cross-Sectional Associations

#### 3.3.1. Age 7

A positive association was observed between a longer EW and BMIz score, with the strongest association seen in model 2; however, effect sizes were small (Table 3). A 13% increase was seen in the odds of being overweight for each hour increase in EW. No associations were seen between EW and any other outcome variables in this age group. WtHR and WC were both positively skewed, with regression residuals being skewed; these were logarithmically transformed.

#### 3.3.2. Age 13

Linear regression showed inverse associations between a longer EW and BMIz, log WtHR, log WC, DBP, and FM in models 1, 2, and 3 (Table 4). Again, effect sizes were small. WtHR and WC were positively skewed, with skewed regression residuals, and so they were logarithmically transformed. These associations were attenuated in model 4, with FM having strongest evidence of an association, albeit with a smaller effect size, whilst log-transformed WC showed weaker evidence of an association in the fully adjusted model.

A longer EW was associated with a 7% reduction in the odds of a high WtHR in models 1, 2, and 3; however, this association was attenuated in model 4. A longer EW was associated with a 6% reduction in the odds of NWO; again, this association was attenuated in model 4. A longer EW was associated with reduced odds of being overweight in model 1, but evidence of this association was weakened in further adjusted models.

### 3.4. Longitudinal Associations

#### 3.4.1. Age 7 to 24

There was weak evidence that a longer EW at age 7 was inversely associated with TC and log triglycerides in model 1 (Table 5). However, associations were attenuated in further adjusted models. BMI, WtHR, WC, and triglycerides were log-transformed due to skewed regression residuals. There was no evidence of any associations between EW and categorical outcomes.

#### 3.4.2. Age 13 to 24

There was strong evidence that a longer EW at age 13 was inversely associated with FM in all models, although the evidence of this association was weaker in model 3 (Table 6). There was weak evidence of an inverse association between a longer EW and BMI; however, this association was attenuated after log transformation. There was no evidence of any association between EW and categorical outcomes.

### 3.5. Sensitivity Analyses

#### 3.5.1. Complete Confounding Data

When model 1 was re-run using only participants with complete confounding data at age 7, the association between a longer EW and BMIz was seen in both models (Table S2), suggesting that this association is not a result of missing data. Similarly, at age 13, these sensitivity analyses supported primary analyses (Table S3). Longitudinal analyses at age 7 did not show any associations in model 1, more closely reflecting the results seen in the further adjusted models (Table S4). Longitudinal analyses at age 13 showed similar associations between models 1 and 2 and supported primary analyses (Table S5).

#### 3.5.2. Complete (3-Day) Diet Diaries

When analyses were restricted to those with complete dietary data over all 3 days at age 7, similar associations were seen between a longer EW and increased BMIz (Table S6), supporting the findings of the primary analyses. In addition, there was some evidence of an association between a longer EW and increased log WtHR and log WC, in contrast to primary analyses, suggesting that 3-day data may better reflect habitual dietary patterns and the primary findings may underestimate associations.

At age 13, this sensitivity analysis again supported primary analyses, with a longer EW having inverse associations with BMIz, log WtHR, log WC, DBP, and FM (Table S7). In addition, stronger inverse associations were seen with log WC and DBP in the fully adjusted model, again suggesting that primary analyses may underestimate associations. A longer EW was associated with reduced odds of both NWO and higher WtHR, supporting primary analyses, with some evidence of this inverse association, with higher WtHR remaining in the fully adjusted model in contrast to primary analyses, further suggesting an underestimation of the associations seen in the primary analyses.

Longitudinally, there was no evidence of any associations between EW at age 7 and outcomes at age 24 (Table S8). EW at age 13 was inversely associated with FM at age 24; however, this was attenuated in the fully adjusted model (Table S9). There was no evidence of any other associations. There was weak evidence that a longer EW at age 13 was associated with reduced odds of NWO at age 24 in model 2, but this association was not seen in the primary analysis. No other associations were seen.

#### 3.5.3. Eating Windows with at Least One Week and One Weekend Day

When restricted to those with both weekday and weekend dietary data, a longer EW at age 7 was positively associated with BMIz in model 2 only (Table S10), supporting primary analyses. There was no evidence of any associations between EW and binary outcomes at age 7. At age 13, sensitivity analyses supported primary findings with inverse associations between a longer EW and log WtHR, log WC, FM, and DBP in model 3 and FM in model 4; however, in contrast to primary analyses, associations were also seen with log WC and DBP in model 4 (Table S11). Logistic regression supported primary findings of a longer EW being associated with reduced odds of NWO and high WtHR. In line with primary analyses, no associations were seen between EW at age 7 and outcomes at age 24 (Table S12). A longer EW at age 13 was associated with reduced FM at age 24 in model 2, supporting primary analysis, with evidence of a weak association in model 3 not seen in primary analysis (Table S13).

## 4. Discussion

To the best of our knowledge, this is the first study examining associations between EW and anthropometric and metabolic outcomes in UK children and adolescents.

### 4.1. Eating Windows

This study found a longer EW at age 13 than at age 7, with considerable variation in individual EW at both ages. First eating times were considerably later on weekends for both ages, with little difference between last eating times on weekdays compared to weekend days; consequently, the EW was longer on weekdays than weekends at both ages.

Adolescence is known to be associated with a shift to a later chronotype, with this shift typically occurring from age 12 onwards [16]. We found later first and last eating times at age 13. This is consistent with the previous literature describing how a move towards a later chronotype in adolescence leads to a later shift in eating times [38]. The differences we found between first eating times on weekdays compared to weekends support the previous literature. Schultz et al. found greater variation in first compared to last eating times at all ages during adolescence [39], whilst Berendsen et al., in a study of older adolescents (aged 13–20), found both first and last mealtimes were later on weekends, although greater variability was seen in first eating times [40]. EW increasing as children get older also supports the previous literature [25,41], likely due to longer waking hours in adolescence than in childhood. A shorter EW on weekends aligns with the previous literature; a study in Spanish children and adolescents, aged 8–13 years, found weekend EW to be shorter, with bigger differences in first than last mealtimes [42].

### 4.2. Cross-Sectional and Longitudinal Associations

A positive association was seen between a longer EW and BMIz score at age 7, whilst at age 13, in contrast to our hypothesis, there were inverse associations between a longer EW and BMIz, log WtHR, log WC, FM, DBP, and the odds of NWO, albeit with small effect sizes. Longitudinally, there were no associations between EW at age 7 and health outcomes at age 24, whilst a longer EW at age 13 was inversely associated with FM at age 24, again with a small effect size, and again in contrast to our hypothesis.

Our findings of a cross-sectional inverse association between a longer EW and BMIz, log WtHR, log WC, FM, DBP, and NWO at age 13, and a longitudinal inverse association between a longer EW at age 13 and FM at age 24, were unexpected. The majority of previous research on associations between EW and health outcomes focuses on adults, and, whilst there are inconsistencies in findings, generally, TLE interventions in adults have demonstrated that a shorter EW was associated with lower body weight, reduced fat mass and blood pressure, and improved glucose and lipid profiles [5,9,10,43]. TLE interventions in CYP have predominantly been pilot studies and, whilst reductions in energy intake and HbA1c have been demonstrated in the TLE group, these have been similar to control groups [12,13,44]. Whilst there has been limited experimental research into child and adolescent populations, a recent cross-sectional analysis found a longer EW was associated with lower BMIz and fat mass index in primary school children (median age 7.9 years) [45]; this contrasts with our finding at age 7 that a longer EW was positively associated with BMIz. We did not have data available to assess associations with FM in this age group; however, the association between a longer EW and reduced FM was the measure most consistently seen across other analyses in our cohort. A cross-sectional study of Russian children aged 13–18 years found that a longer EW was weakly associated with reduced BMI percentiles [46], which is similar to our findings at age 13. Furthermore, a recent cohort study found that a shorter EW was associated with higher insulin resistance in children aged 8 to 15 years [47]. Again, we did not have data available to assess this in our cohort. The authors of that study suggested that the increased insulin sensitivity associated with a longer EW may be due to the beneficial effects of eating breakfast. It is possible that the association between a longer EW and reduced anthropometric measures seen in our study is also a result of breakfast eating. The beneficial effects on body composition seen in adolescents who eat breakfast are well-documented [48,49], and it is plausible that breakfast eaters will have a longer EW. However, in contrast to our study, a cross-sectional study of Spanish children aged 8–13 years did not find any associations between EW and anthropometric measures, although they did find that a longer EW was associated with increased glucose and LDL-C levels and reduced triglyceride levels [42]. Again, none of these measures were available in our cohort at ages 7 or 13.

### 4.3. Potential Mechanisms for Associations Between EW and Outcomes

The unexpected results of a longer EW at age 13 being associated with better outcomes, whilst similar to some recent studies in similar age groups, contrast with the larger volume of adult research. The causative mechanism behind these unexpected associations is not fully understood, but it is possible there is a dietary factor contributing to a shorter EW that is driving these results; further research on CYP is needed. Breakfast skipping, with those who skipped breakfast likely to have a shorter EW, may offer an explanation. Missing breakfast is known to be associated with less favourable body composition, potentially due to altered hormonal responses to meals consumed later in the day and reduced DIT with later meals [2,3,4]. Studies demonstrate that CYP who skip breakfast have increased body weight, worse lipid profiles, higher blood pressure, and increased insulin resistance [48,49,50]. Alternatively, physical activity levels may be associated with EW in childhood and adolescence; it is possible that those with a longer EW were more physically active. However, this was not a confounder that we were able to adjust for in our analyses. These unknowns suggest further research is needed to ascertain what dietary or lifestyle habits are associated with length of EW and whether there are specific elements of child or adolescent diets within a specific EW that are linked to less favourable outcomes.

### 4.4. Strengths and Limitations

Our study has some strengths and limitations. Its main strength is the longitudinal design, with follow-up over many years enabling assessments to be made of the effects of dietary habits over time whilst adjusting for confounders. Additionally, the sample size is large. Whilst attrition, typically seen in longitudinal studies, reduced the sample sizes over time, the smallest samples at age 24 were still in excess of 2000 participants.

However, this study is not without limitations. First, the diet diaries were not designed specifically to assess timing of eating; consequently, eating times were not always recorded. This loss of data, with missing first or last times meaning an eating window was unable to be calculated, may have impacted the results. Additionally, diet diaries did not record both the start and finish time of eating, meaning that the calculated EW may be an underestimate. Furthermore, the diet diaries relied on self-reports, which may be prone to reporting errors; however, the prospective nature of the diaries should aid accuracy by limiting recall bias.

Second, ALSPAC participants were all from a limited geographical area, meaning the sample may not be generalisable to the rest of the UK or worldwide. Additionally, there were differences between CYP who did and did not attend clinics, with non-attenders having mothers who were younger, less educated, and of lower household social class. This selection bias may have impacted the results seen, and these factors, plus the lack of ethnic diversity of our sample, may limit the generalisability of our results.

Third, missing data, as is typical of longitudinal studies, may have impacted the results. However, our sensitivity analyses suggested that our results were robust to missing data from confounding variables or missing dietary information.

Fourth, there are many factors that can affect eating habits; body composition and metabolic health that were not considered in this research, including weight regulating hormones, such as leptin and ghrelin, psychological and emotional factors, physical health conditions, and physical activity levels. There is potential that these could have impacted either EW or health outcomes, and these should be considered in future research.

Fifth, the observational nature of our study means that whilst we found associations between EW and outcome measures, we cannot be sure that the EW was causative. Additionally, the effect sizes we found were small, limiting the clinical significance of our findings; however, the importance of these inverse associations in adolescents remains.

It should be noted that our DAGs suggested that diet quality and energy intake were mediators and should therefore not be included as confounders in our regression models. However, despite basing this decision on previous evidence and expert knowledge, it is possible that the association between EW and diet quality or energy intake could be bi-directional. For this reason, plus the importance of energy intake and diet quality for our outcomes, we felt it necessary to include them in a final model, with the caveat that this may be overfitted. We did find that the inclusion of these variables attenuated the associations seen at all timepoints, adding strength to our assumptions that these may be mediators and that our fully adjusted model was overfitted.

Due to the longitudinal nature of the ALSPAC cohort, dietary data collected in childhood or adolescence are now approximately 20 years old. Changes to dietary intake and the food landscape may mean that young people’s diets have changed. Thus, further research into contemporary UK populations is needed.

## 5. Conclusions

A longer EW was associated with increased BMIz at age 7 and reduced BMIz, log WtHR, log WC, FM, DBP, and NWO at age 13. Additionally, a longer EW at age 13 was associated with reduced FM at age 24, but with small effect sizes limiting clinical importance. Therefore, interventions designed to shorten EWs in children and young people are not supported by this research. However, these findings, alongside the contrasting results across previous research, demonstrate the complexity of the interplay between diet and health, highlighting the need for further chrono-nutritional research. The relationships between meal timings and health in childhood and adolescence, and how these can impact health into adulthood, need further study. Future research should aim to understand which element of a longer EW is driving the inverse associations seen between EW and health outcomes, whilst replication in well-designed longitudinal studies in other populations is also required.

## Figures and Tables

**Table 1:** Characteristics of participants with both dietary and other data at each timepoint.

	Age 7 (N = 4835)	Age 13 (N = 4719)	Age 24 (N = 2568)
Age (years)	7.5 (0.3)	13.8 (0.2)	24.0 (0.8)
Sex			
Female	2607 (54%)	2469 (52%)	1622 (63%)
Male	2228 (46%)	2250 (48%)	946 (37%)
Ethnicity			
White	4147 (96.4%)	4163 (96.4%)	2295 (96.3%)
Non-white	153 (3.6%)	155 (3.6%)	87 (3.7%)
BMI z score	0.1 (1.0)	0.3 (1.1)	-
BMI (kg/m^2^)	-	-	23.6 (21.4, 26.9)
WC (cm)	55.5 (53.0, 58.7)	70.0 (65.7, 76.0)	78.7 (72.1, 87.5)
WtHR	0.44 (0.42, 0.46)	0.43 (0.40, 0.46)	0.46 (0.42, 0.50)
FM (%)	-	24.4 (10.2)	31.2 (9.0)
NWO			
Yes	-	1096 (23.6%)	821 (33.2%)
No	-	3546 (76.4%)	1652 (66.8%)
SBP (mmHg)	99.1 (9.2)	106.6 (9.4)	116.0 (11.5)
DBP (mmHg)	56.5 (6.7)	57.6 (6.0)	67.0 (8.1)
TC (mmol/L)	-	-	4.4 (0.8)
HDL-C (mmol/L)	-	-	1.6 (0.4)
LDL-C (mmol/L)	-	-	2.4 (0.8)
Triglycerides (mmol/L)	-	-	0.8 (0.7, 1.1)
FG (mmol/L)	-	-	5.3 (0.6)

Data are means (standard deviation) or medians (interquartile range) for continuous variables and number (percent) for categorical variables. BMI: body mass index; WC: waist circumference; WtHR: waist to height ratio; FM: fat mass; NWO: normal weight obesity; SBP: systolic blood pressure; DBP: diastolic blood pressure; TC: total cholesterol; HDL-C: high-density lipoprotein cholesterol; LDL-C: low-density lipoprotein cholesterol; FG: fasting glucose.

**Table 2:** Eating times and eating windows at ages 7 and 13.

	Age 7		Age 13	
	**N**	**Time**	**N**	**Time**
All diaries—first	4851	8.12 (0.6)	4741	8.68 (1.3)
All diaries—last	4851	19.05 (1.0)	4741	19.79 (1.4)
Weekday—first	4793	7.92 (0.6)	4426	8.34 (1.4)
Weekday—last	4793	19.03 (1.1)	4426	19.79 (1.5)
Weekend—first	3742	8.60 (0.9)	2669	8.68 (1.3)
Weekend—last	3742	19.08 (1.2)	2669	19.79 (1.4)
	**N**	**Hours**	**N**	**Hours**
Eating window (all days)	4848	10.9 (1.1)	4741	11.1 (1.8)
Weekday eating window	4793	11.1 (1.2)	4426	11.5 (1.9)
Weekend eating window	3742	10.5 (1.4)	2669	10.3 (2.0)

N: Number. Data are means (standard deviation).

**Table 3:** Associations between eating window (hours) and outcomes at age 7.

	Model 1			Model 2			Model 3		
	**β**	**95% CI**	**P**	**β**	**95% CI**	**P**	**β**	**95% CI**	**P**
BMIz	0.027	−0.001, 0.055	0.06	0.041	0.011, 0.071	0.01	0.012	−0.019, 0.043	0.45
Log WtHR	0.001	−0.001, 0.003	0.44	0.002	−0.000, 0.004	0.10	0.002	−0.001, 0.004	0.19
Log WC	0.001	−0.001, 0.003	0.36	0.002	−0.000, 0.005	0.09	−0.000	−0.003, 0.002	0.87
SBP (mmHg)	0.055	−0.191, 0.301	0.66	0.041	−0.235, 0.317	0.77	−0.218	−0.501, 0.065	0.13
DBP (mmHg)	0.003	−0.175, 0.181	0.97	−0.064	−0.265, 0.136	0.53	−0.151	−0.358, 0.055	0.15
	**OR**	**95% CI**	**P**	**OR**	**95% CI**	**P**	**OR**	**95% CI**	**P**
WtHR									
Healthy	Ref.			Ref.			Ref.		
High	1.04	0.94, 1.15	0.46	1.07	0.95, 1.20	0.28	1.09	0.97, 1.24	0.15
BMIz									
Healthy	Ref			Ref.			Ref.		
Overweight	1.06	0.97, 1.15	0.19	1.13	1.02, 1.24	0.02	1.09	0.98, 1.21	0.10

β: regression coefficient; CI: confidence interval; P: *p*-value; BMIz: body mass index z-score; Log: logarithmic transformation; WtHR: waist to height ratio; WC: waist circumference; SBP: systolic blood pressure; DBP: diastolic blood pressure; OR: odds ratio; Ref: reference category; WtHR: healthy ≤ 0.49, high ≥ 0.50; BMIz: healthy ≤ 1.33 standard deviation (SD), overweight ≥ 1.34 SD. Model 1 was adjusted for age and sex. Model 2 was additionally adjusted for mother’s age, mother’s BMI, mother’s education level, and household social class. Model 3 was additionally adjusted for diet quality scores and energy intake.

**Table 4:** Associations between eating window (hours) and outcomes at age 13.

	Model 1			Model 2			Model 3			Model 4		
	**β**	**95% CI**	**P**	**β**	**95% CI**	**P**	**β**	**95% CI**	**P**	**β**	**95% CI**	**P**
BMIz	−0.028	−0.046, −0.010	0.003	−0.023	−0.042, −0.004	0.02	−0.026	−0.046, −0.006	0.01	−0.018	−0.039, 0.002	0.08
Log WtHR	−0.003	−0.005, −0.001	<0.001	−0.003	−0.005, −0.001	0.005	−0.003	−0.005, −0.001	0.004	−0.001	−0.004, 0.001	0.17
Log WC	−0.003	−0.005, −0.001	0.002	−0.002	−0.005, −0.000	0.02	−0.003	−0.005, −0.001	0.007	−0.002	−0.004, −0.000	0.04
FM (%)	−0.404	−0.547, −0.261	<0.001	−0.375	−0.529, −0.222	<0.001	−0.447	−0.607, −0.286	<0.001	−0.253	−0.417, −0.089	0.002
SBP (mmHg)	−0.120	−0.279, 0.038	0.14	−0.136	−0.305, 0.034	0.12	−0.102	−0.290, 0.086	0.29	−0.179	−0.374, 0.016	0.07
DBP (mmHg)	−0.136	−0.237, −0.035	0.008	−0.172	−0.282, −0.061	0.002	−0.132	−0.254, −0.009	0.04	−0.120	−0.247, 0.007	0.06
	**OR**	**95% CI**	**P**	**OR**	**95% CI**	**P**	**OR**	**95% CI**	**P**	**OR**	**95% CI**	**P**
WtHR												
Healthy	Ref.			Ref.			Ref.			Ref.		
High	0.92	0.88, 0.97	<0.001	0.93	0.89, 0.98	0.01	0.93	0.88, 0.99	0.02	0.97	0.91, 1.03	0.36
BMIz												
Healthy	Ref.			Ref.			Ref.					
Overweight	0.96	0.92, 0.99	0.03	0.96	0.92, 1.01	0.12	0.96	0.91, 1.01	0.10	0.98	0.92, 1.03	0.43
NWO												
No	Ref.			Ref.			Ref.			Ref.		
Yes	0.96	0.93, 1.00	0.04	0.96	0.92, 1.00	0.08	0.94	0.90, 0.99	0.01	0.98	0.93, 1.03	0.41

β: regression coefficient; CI: confidence interval; P: *p*-value; BMIz: body mass index z-score; Log: logarithmic transformation; WtHR: waist to height ratio; WC: waist circumference; FM: fat mass; SBP: systolic blood pressure; DBP: diastolic blood pressure; OR: odds ratio; Ref: reference category; NWO: normal weight obesity; WtHR: healthy ≤ 0.49, high ≥ 0.50; BMIz: healthy ≤ 1.33 standard deviation (SD), overweight ≥ 1.34 SD. Model 1 was adjusted for age and sex. Model 2 was additionally adjusted for pubertal status. Model 3 was additionally adjusted for mother’s age, mother’s BMI, mother’s education level, and household social class. Model 4 was additionally adjusted for diet quality scores and energy intake.

**Table 5:** Associations between eating window (hours) at age 7 and outcomes at age 24.

	Model 1			Model 2			Model 3		
	**β**	**95% CI**	**P**	**β**	**95% CI**	**P**	**β**	**95% CI**	**P**
Log BMI	−0.002	−0.009, 0.005	0.62	0.002	−0.005, 0.009	0.59	−0.003	−0.010, 0.005	0.48
Log WtHR	−0.002	−0.008, 0.003	0.36	−0.000	−0.006, 0.005	0.96	−0.002	−0.007, 0.004	0.49
Log WC	−0.003	−0.008, 0.003	0.33	−0.000	−0.006, 0.005	0.90	−0.004	−0.009, 0.002	0.20
FM (%)	−0.208	−0.493, 0.077	0.15	−0.086	−0.386, 0.214	0.57	−0.186	−0.492, 0.121	0.23
SBP (mmHg)	−0.243	−0.620, 0.134	0.21	−0.159	−0.580, 0.262	0.46	−0.368	−0.799, 0.062	0.09
DBP (mmHg)	−0.113	−0.412, 0.186	0.46	−0.062	−0.395, 0.270	0.71	−0.129	−0.471, 0.212	0.46
TC (mmol/L)	−0.038	−0.072,−0.004	0.03	−0.029	−0.066, 0.008	0.13	−0.025	−0.064, 0.013	0.19
HDL-C (mmol/L)	0.002	−0.014, 0.019	0.81	−0.001	−0.019, 0.017	0.94	0.001	−0.018, 0.020	0.92
LDL-C (mmol/L)	−0.027	−0.057, 0.004	0.09	−0.020	−0.054, 0.013	0.24	−0.019	−0.053, 0.016	0.28
Log trig	−0.022	−0.039,−0.005	0.01	−0.013	−0.032, 0.005	0.17	−0.013	−0.032, 0.007	0.20
FG (mmol/L)	−0.003	−0.028, 0.021	0.78	0.001	−0.027, 0.030	0.92	0.006	−0.023, 0.035	0.67
	**OR**	**95% CI**	**P**	**OR**	**95% CI**	**P**	**OR**	**95% CI**	**P**
WtHR									
Healthy	Ref.			Ref.			Ref.		
High	0.96	0.88, 1.04	0.31	0.99	0.90, 1.09	0.88	0.96	0.87, 1.07	0.49
BMI									
Healthy	Ref.			Ref.			Ref.		
Overweight	0.95	0.88, 1.02	0.18	0.99	0.90, 1.08	0.79	0.94	0.86, 1.04	0.22
NWO									
No	Ref.			Ref.			Ref.		
Yes	1.04	0.96, 1.12	0.38	1.01	0.93, 1.11	0.76	1.04	0.95, 1.14	0.45

β: regression coefficient; CI: confidence interval; P: *p*-value; Log: logarithmic transformation; BMI: body mass index; WtHR: waist to height ratio; WC: waist circumference; FM: fat mass; SBP: systolic blood pressure; DBP: diastolic blood pressure; TC: total cholesterol; HDL-C: high-density lipoprotein cholesterol; LDL-C: low-density lipoprotein cholesterol; Trig: triglycerides; FG: fasting glucose; OR: odds ratio; Ref: reference category; NWO: normal weight obesity; WtHR: healthy ≤ 0.49, high ≥ 0.50; BMI: healthy ≤ 24.9 kg/m^2^, overweight ≥ 25 kg/m^2^. Model 1 was adjusted for age and sex. Model 2 was additionally adjusted for mother’s age, mother’s BMI, mother’s education level, and household social class. Model 3 was additionally adjusted for diet quality scores and energy intake.

**Table 6:** Associations between eating window (hours) at age 13 and outcomes at age 24.

	Model 1			Model 2			Model 3		
	**β**	**95% CI**	**P**	**β**	**95% CI**	**P**	**β**	**95% CI**	**P**
Log BMI	−0.004	−0.008, −0.000	0.05	−0.004	−0.008, 0.000	0.08	−0.003	−0.007, 0.001	0.15
Log WtHR	−0.003	−0.006, 0.000	0.09	−0.002	−0.005, 0.001	0.16	−0.001	−0.004, 0.002	0.52
Log WC	−0.002	−0.005, 0.001	0.16	−0.002	−0.005, 0.001	0.25	−0.001	−0.005, 0.002	0.41
FM (%)	−0.299	−0.471, −0.127	<0.001	−0.307	−0.487, −0.127	<0.001	−0.192	−0.377, −0.007	0.04
SBP (mmHg)	−0.008	−0.233, 0.217	0.95	0.119	−0.130, 0.368	0.35	0.104	−0.153, 0.361	0.43
DBP (mmHg)	−0.056	−0.233, 0.121	0.53	0.017	−0.178, 0.212	0.87	0.068	−0.133, 0.270	0.51
TC (mmol/L)	−0.008	−0.029, 0.012	0.43	−0.004	−0.027, 0.018	0.70	−0.003	−0.026, 0.021	0.83
HDL-C (mmol/L)	0.005	−0.005, 0.015	0.29	0.005	−0.006, 0.016	0.37	0.004	−0.007, 0.015	0.47
LDL-C (mmol/L)	−0.008	−0.026, 0.011	0.42	−0.005	−0.026, 0.015	0.63	−0.003	−0.024, 0.018	0.80
Log trig	−0.009	−0.020, 0.001	0.08	−0.008	−0.020, 0.004	0.18	−0.007	−0.019, 0.005	0.28
FG (mmol/L)	−0.016	−0.034, 0.002	0.08	0.001	−0.016, 0.017	0.94	−0.003	−0.020, 0.013	0.70
	**OR**	**95% CI**	**P**	**OR**	**95% C**	**P**	**OR**	**95% CI**	**P**
WtHR									
Healthy	Ref.			Ref.			Ref.		
High	0.98	0.94, 1.04	0.55	0.98	0.92, 1.04	0.50	1.00	0.94, 1.06	0.98
BMI									
Healthy	Ref.			Ref.			Ref.		
Overweight	0.97	0.93, 1.02	0.27	0.97	0.92, 1.02	0.28	0.98	0.93, 1.04	0.50
NWO									
No	Ref.			Ref.			Ref.		
Yes	0.97	0.92, 1.02	0.22	0.98	0.92, 1.03	0.37	0.98	0.93, 1.04	0.58

β: regression coefficient; CI: confidence interval; P: *p*-value; Log: logarithmic transformation; BMI: body mass index; WtHR: waist to height ratio; WC: waist circumference; FM: fat mass; SBP: systolic blood pressure; DBP: diastolic blood pressure; TC: total cholesterol; HDL-C: high-density lipoprotein cholesterol; LDL-C: low-density lipoprotein cholesterol; Trig: triglycerides; FG: fasting glucose; OR: odds ratio; Ref: reference category; NWO: normal weight obesity; WtHR: healthy ≤ 0.49, high ≥ 0.50; BMI: healthy ≤ 24.9 kg/m^2^, overweight ≥ 25 kg/m^2^. Model 1 was adjusted for age and sex. Model 2 was additionally adjusted for mother’s age, mother’s BMI, mother’s education level, and household social class. Model 3 was additionally adjusted for diet quality scores and energy intake.

## Data Availability

The informed consent obtained from ALSPAC participants does not allow for the data to be made freely available through any third-party-maintained public repository. However, data used for this submission can be made available upon request to the ALSPAC Executive. The ALSPAC data management plan describes in detail the policy regarding data sharing, which occurs through a system of managed open access. Full instructions for applying for data access can be found here: http://www.bristol.ac.uk/alspac/researchers/access/ (accessed 24 March 2025). The ALSPAC study website contains details of all of the data that are available (http://www.bristol.ac.uk/alspac/researchers/our-data/) (accessed 24 March 2025).

## Supplementary Materials

The following supporting information can be downloaded at https://www.mdpi.com/article/10.3390/nu17172856/s1, Table S1: Characteristics of clinic attendees and non-attendees; Table S2: Sensitivity analysis with complete dietary data. Associations between eating window (hours) and outcomes at age 7; Table S3: Sensitivity analysis with complete dietary data. Associations between eating window (hours) and outcomes at age 13; Table S4: Sensitivity analysis with complete dietary data. Longitudinal associations between eating window (hours) at age 7 and outcomes at age 24; Table S5: Sensitivity analysis with complete dietary data. Longitudinal associations between eating window (hours) at age 13 and outcomes at age 24; Table S6: Sensitivity analysis with both weekday and weekend dietary data. Associations between eating window (hours) and outcomes at age 7; Table S7: Sensitivity analysis with weekday and weekend dietary data. Associations between eating window (hours) and outcomes at age 13; Table S8: Sensitivity analysis with weekday and weekend dietary data. Longitudinal associations between eating window (hours) at age 7 and outcomes at age 24; Table S9: Sensitivity analysis with weekday and weekend dietary data. Longitudinal associations between eating window (hours) at age 13 and outcomes at age 24; Table S10: Sensitivity analysis using complete confounder data. Associations between eating window (hours) and outcomes at age 7 using only participants from model 2; Table S11: Sensitivity analysis using complete confounder data. Associations between eating window (hours) and outcomes at age 13 using only participants from model 2; Table S12: Sensitivity analysis using complete confounder data. Longitudinal associations between eating window (hours) at age 7 and outcomes at age 24 using only participants from model 2; Table S13: Sensitivity analysis using complete confounder data. Longitudinal associations between eating window (hours) at age 13 and outcomes at age 24 using only participants from model 2.

## Abbreviations

The following abbreviations are used in this manuscript:

ALSPAC	Avon Longitudinal Study of Parents and Children
β	beta coefficient
BMIz	body mass index z-score
CI	confidence interval
CYP	children and young people
DAG	directed acyclic graph
DBP	diastolic blood pressure
DXA	dual-energy X-ray absorptiometry
EW	eating window
FM	fat mass
HDL-C	high-density lipoprotein cholesterol
IQR	interquartile range
LDL-C	low-density lipoprotein cholesterol
N	number
NOW	normal weight obesity
SBP	systolic blood pressure
SD	standard deviation
TC	total cholesterol
TLE	time-limited eating
WC	waist circumference
WtHR	waist to height ratio
YP	young people
