# Supplementary material for: Associations Between Eating Windows and Health Outcomes in Children and Adolescents from the ALSPAC Cohort
Source: Nutrients. 2025 Sep 3;17(17):2856. doi: 10.3390/nu17172856 (PMC12430200; doi:10.3390/nu17172856)
Supplement: Supplementary file 1 [file nutrients-17-02856-s001.zip › nutrients-3782330-supplementary.pdf]

Table S1. Characteristics of clinic attendees and non-attendees

	Age 7			Age 13			Age 24		
	Attendees	Non-attendees	p-value	Attendees	Non-attendees	p-value	Attendees	Non-attendees	p-value
N	8,274 (52.9%)	7,377 (47.1%)		6,130 (39.2%)	9,521 (60.8%)		4,017 (25.7%)	11,634 (74.3%)	
Sex									
Male	4,193 (50.7%)	3,490 (51.7%)	0.23	3,008 (49.1%)	4,675 (52.5%)	<0.001	1,503 (37.4%)	6,180 (56.1%)	<0.001
Female	4,081 (49.3%)	3,265 (48.3%)		3,122 (50.9%)	4,224 (47.5%)		2,514 (62.6%)	4,832 (43.9%)	
Maternal age	29.0 (4.6)	26.7 (5.1)	<0.001	29.1 (4.5)	27.2 (5.1)	<0.001	29.4 (4.6)	27.5 (5.0)	<0.001
Maternal education									
None/CSE	729 (10.1%)	1,020 (22.9%)	<0.001	511 (9.4%)	1,238 (19.9%)	<0.001	252 (7.2%)	1,497 (18.3%)	<0.001
Vocational	650 (9.0%)	579 (13.0%)		460 (8.4%)	769 (12.3%)		269 (7.7%)	960 (11.7%)	
O Level	2,661 (36.8%)	1,657 (37.2%)		1,989 (36.4%)	2,329 (37.4%)		1,220 (34.7%)	3,098 (37.9%)	
A level	1,993 (27.5%)	799 (17.9%)		1,542 (28.2%)	1,250 (20.1%)		1,035 (29.5%)	1,757 (21.5%)	
Degree	1,206 (16.7%)	400 (9.0%)		958 (17.5%)	648 (10.4%)		737 (21.0%)	869 (10.6%)	
Social class									
I	1,123 (15.7%)	413 (9.4%)	<0.001	898 (16.6%)	638 (10.4%)	<0.001	673 (19.4%)	863 (10.7%)	<0.001
II	3,227 (45.0%)	1,598 (36.5%)		2,459 (45.5%)	2,366 (38.5%)		1,586 (45.8%)	3,239 (40.1%)	
III (non-manual)	1,776 (24.8%)	1,168 (26.7%)		1,321 (24.4%)	1,623 (26.4%)		803 (23.2%)	2,141 (26.5%)	
III (manual)	745 (10.4%)	817 (18.7%)		515 (9.5%)	1,047 (17.0%)		292 (8.4%)	1,270 (15.7%)	
IV	264 (3.7%)	318 (7.3%)		189 (3.5%)	393 (6.4%)		101 (2.9%)	481 (5.9%)	
V	36 (0.5%)	65 (1.5%)		22 (0.4%)	79 (1.3%)		10 (0.3%)	91 (1.1%)	

Numbers given are n (%) for categorical variables and mean (SD) for continuous variables. N: number, SD: standard deviation. Social class categories are National Statistics Socioeconomic Classification (NS-SEC) categories, highest across both parents.

Table S2. Sensitivity analysis using complete confounder data. Associations between eating window (hours) and outcomes at age 7 using only participants from model 2

	Model 1			Model 2		
	β	95% CI	P	β	95% CI	P
BMIz	0.038	0.007, 0.070	0.02	0.041	0.011, 0.071	0.01
Log WtHR	0.002	-0.001, 0.004	0.19	0.002	-0.000, 0.004	0.10
Log WC	0.002	-0.001, 0.005	0.12	0.002	-0.000, 0.005	0.09
SBP (mmHg)	0.024	-0.252, 0.300	0.87	0.041	-0.235, 0.317	0.77
DBP (mmHg)	-0.079	-0.278, 0.121	0.44	-0.064	-0.265, 0.136	0.53
	OR	95% CI	P	OR	95% CI	P
WtHR						
Healthy	Ref.			Ref.		
High	1.05	0.93, 1.18	0.42	1.07	0.95, 1.20	0.28
BMIz						
Healthy	Ref.			Ref.		
Overweight	1.12	1.01, 1.23	0.02	1.13	1.02, 1.24	0.02

β: beta regression coefficient, CI: confidence interval, P: p-value, BMIz: body mass index z-score, Log: logarithmic transformation, WtHR: waist to height ratio, WC: waist circumference, SBP: systolic blood pressure, DBP: diastolic blood pressure, OR: odds ratio.

Note: WtHR: Healthy ≤0.49, High ≥0.50; BMIz: Healthy ≤1.33 standard deviation (SD), Overweight ≥1.34 SD.

Model 1 adjusted for child's age and child's sex. Model 2 additionally adjusted for mother's age, mother's BMI, mother's education level and household social class.

Table S3. Sensitivity analysis using complete confounder data. Associations between eating window (hours) and outcomes at age 13 using only participants from model 2

	Model 1			Model 2		
	β	95% CI	P	β	95% CI	P
BMIz	-0.031	-0.053, -0.010	0.004	-0.026	-0.046, -0.006	0.01
Log WtHR	-0.004	-0.006, -0.001	0.001	-0.003	-0.005, -0.001	0.004
Log WC	-0.003	-0.006, -0.001	0.002	-0.003	-0.005, -0.001	0.007
FM (%)	-0.475	-0.644, -0.305	<0.001	-0.447	-0.607, -0.286	<0.001
SBP (mmHg)	-0.127	-0.320, 0.066	0.20	-0.102	-0.290, 0.086	0.29
DBP (mmHg)	-0.144	-0.266, -0.021	0.02	-0.132	-0.254, -0.009	0.04

	OR	95% CI	P	OR	95% CI	P
WtHR						
Healthy						
High	0.92	0.87, 0.98	0.008	0.93	0.87, 0.99	0.02
BMIz						
Healthy						
Overweight	0.95	0.90, 1.00	0.05	0.96	0.91, 1.01	0.09
NWO						
No						
Yes	0.94	0.90, 0.99	0.02	0.94	0.90, 0.99	0.01

β: beta regression coefficient, CI: confidence interval, P: p-value, BMIz: body mass index z-score, Log: logarithmic transformation, WtHR: waist to height ratio, WC: waist circumference, FM: fat mass, SBP: systolic blood pressure, DBP: diastolic blood pressure, OR: odds ratio, NWO: normal weight obesity.

Note: WtHR: Healthy ≤0.49, High ≥0.50; BMIz: Healthy ≤1.33 standard deviation (SD), Overweight ≥1.34 SD.

Model 1 adjusted for child's age and child's sex. Model 2 additionally adjusted for mother's age, mother's BMI, mother's education level and household social class.

Table S4. Sensitivity analysis using complete confounder data. Longitudinal associations between eating window (hours) at age 7 and outcomes at age 24 using only participants from model 2

	Model 1			Model 2		
	β	95% CI	P	β	95% CI	P
Log BMI	0.000	-0.007, 0.008	0.99	0.002	-0.005, 0.009	0.59
Log WtHR	-0.002	-0.007, 0.004	0.57	-0.000	-0.006, 0.005	0.96
Log WC	-0.002	-0.007, 0.004	0.58	-0.000	-0.006, 0.005	0.90
FM (%)	-0.147	-0.459, 0.166	0.36	-0.086	-0.386, 0.214	0.57
SBP (mmol/L)	-0.199	-0.620, 0.222	0.35	-0.159	-0.580, 0.262	0.46
DBP (mmol/L)	-0.114	-0.448, 0.220	0.50	-0.062	-0.395, 0.270	0.71
TC (mmol/L)	-0.033	-0.070, 0.004	0.08	-0.029	-0.066, 0.008	0.13
HDL-C (mmol/L)	0.001	-0.017, 0.019	0.90	-0.001	-0.019, 0.017	0.94
LDL-C (mmol/L)	-0.025	-0.058, 0.009	0.15	-0.020	-0.054, 0.013	0.24
Log triglycerides	-0.016	-0.035, 0.003	0.09	-0.013	-0.032, 0.005	0.17
FG (mmol/L)	0.002	-0.026, 0.030	0.87	0.001	-0.027, 0.030	0.92

	OR	95% CI	P	OR	95% CI	P
WtHR						
Healthy	Ref.			Ref.		
High	0.97	0.88, 1.06	0.49	0.99	0.90, 1.09	0.88
BMI						
Healthy	Ref.			Ref.		
Overweight	0.97	0.89, 1.06	0.51	0.99	0.90, 1.08	0.79
NWO						
No	Ref.			Ref.		
Yes	1.02	0.93, 1.11	0.72	1.01	0.93, 1.11	0.76

β: beta regression coefficient, CI: confidence interval, P: p-value, Log: logarithmic transformation, BMI: body mass index, WtHR: waist to height ratio, WC: waist circumference, FM: fat mass, SBP: systolic blood pressure, DBP: diastolic blood pressure, TC: total cholesterol, HDL-C: high density lipoprotein cholesterol, LDL-C: low density lipoprotein cholesterol, FG: fasting glucose, OR: odds ratio, NWO: normal weight obesity.

Note: WtHR: Healthy ≤0.49, High ≥0.50; BMI: Healthy ≤24.9 kg/m^2, Overweight ≥25 kg/m^2.

Model 1 adjusted for child's age and child's sex. Model 2 additionally adjusted for mother's age, mother's BMI, mother's education level and household social class.

Table S5. Sensitivity analysis using complete confounder data. Longitudinal associations between eating window (hours) at age 13 and outcomes at age 24 using only participants from model 2

	Model 1			Model 2		
	β	95% CI	P	β	95% CI	P
Log BMI	-0.004	-0.009, 0.000	0.07	-0.004	-0.008, 0.000	0.08
Log WtHR	-0.002	-0.006, 0.001	0.15	-0.002	-0.005, 0.001	0.16
Log WC	-0.002	-0.005, 0.001	0.22	-0.002	-0.005, 0.001	0.25
FM (%)	-0.327	-0.515, -0.139	<0.001	-0.307	-0.487, -0.127	<0.001
SBP (mmHg)	0.087	-0.163, 0.337	0.50	0.119	-0.130, 0.368	0.35
DBP (mmHg)	-0.004	-0.200, 0.192	0.97	0.017	-0.178, 0.212	0.87
TC (mmol/L)	-0.004	-0.026, 0.019	0.76	-0.004	-0.027, 0.018	0.70
HDL-C (mmol/L)	0.006	-0.005, 0.017	0.28	0.005	-0.006, 0.016	0.37
LDL-C (mmol/L)	-0.005	-0.025, 0.016	0.65	-0.005	-0.026, 0.015	0.63
Log triglycerides	-0.009	-0.020, 0.003	0.14	-0.008	-0.020, 0.004	0.18
FG (mmol/L)	0.000	-0.016, 0.016	1.00	0.001	-0.016, 0.017	0.94
	**OR**	**95% CI**	**P**	**OR**	**95% CI**	**P**
WtHR						
Healthy	Ref.			Ref.		
High	0.98	0.92, 1.04	0.44	0.98	0.92, 1.04	0.50
BMI						
Healthy	Ref.			Ref.		
Overweight	0.97	0.92, 1.02	0.28	0.97	0.92, 1.02	0.28
NWO						
No	Ref.			Ref.		
Yes	0.98	0.92, 1.03	0.36	0.98	0.92, 1.03	0.37

β*: beta regression coefficient, CI: confidence interval, P: p-value, Log: logarithmic transformation, BMI: body mass index, WtHR: waist to height ratio, WC: waist circumference, FM: fat mass, SBP: systolic blood pressure, DBP: diastolic blood pressure, TC: total cholesterol, HDL-C: high density lipoprotein cholesterol, LDL-C: low density lipoprotein cholesterol, FG: fasting glucose, OR: odds ratio, NWO: normal weight obesity.*

Note: WtHR: Healthy ≤0.49, High ≥0.50; BMI: Healthy ≤24.9 kg/m^2, Overweight ≥25 kg/m^2.

Model 1 adjusted for child's age and child's sex. Model 2 additionally adjusted for mother's age, mother's BMI, mother's education level and household social class.

Table S6. Sensitivity analysis with complete (3 days) dietary data. Associations between eating window (hours) and outcomes at age 7

	Model 2			Model 3		
	β	95% CI	P	β	95% CI	P
BMIz	0.052	0.018, 0.087	0.003	0.021	-0.014, 0.055	0.25
Log WtHR	0.003	0.000, 0.005	0.02	0.003	-0.000, 0.005	0.05
Log WC (cm)	0.003	0.000, 0.006	0.03	0.000	-0.002, 0.003	0.73
SBP (mmHg)	0.078	-0.236, 0.393	0.62	-0.185	-0.506, 0.136	0.26
DBP (mmHg)	-0.011	-0.239, 0.218	0.93	-0.114	-0.349, 0.120	0.34

	OR	95% CI	P	OR	95% CI	P
WtHR						
Healthy	Ref.			Ref.		
High	1.10	0.96, 1.26	0.16	1.13	0.98, 1.30	0.09
BMIz						
Healthy	Ref.			Ref.		
Overweight	1.14	1.02, 1.28	0.02	1.10	0.98, 1.24	0.10

β: beta regression coefficient, CI: confidence interval, P: p-value, BMIz: body mass index z-score, Log: logarithmic transformation, WtHR: waist to height ratio, WC: waist circumference, SBP: systolic blood pressure, DBP: diastolic blood pressure, OR: odds ratio.

Note: WtHR: Healthy ≤0.49, High ≥0.50; BMIz: Healthy ≤1.33 standard deviation (SD), Overweight ≥1.34 SD.

Model 2 adjusted for for child's age, child's sex, mother's age, mother's BMI, mother's education level and household social class. Model 3 additionally adjusted for diet quality scores and energy intake.

Table S7. Sensitivity analysis with complete (3 days) dietary data. Associations between eating window (hours) and outcomes at age 13

	Model 3			Model 4		
	β	95% CI	P	β	95% CI	P
BMIz	-0.039	-0.069, -0.008	0.01	-0.032	-0.063, 0.000	0.05
Log WtHR	-0.004	-0.007, -0.001	0.006	-0.003	-0.006, 0.000	0.07
Log WC (cm)	-0.004	-0.007, -0.001	0.01	-0.004	-0.007, -0.001	0.02
FM (%)	-0.566	-0.807, -0.325	<0.001	-0.342	-0.590, -0.094	0.007
SBP (mmHg)	-0.156	-0.436, 0.124	0.27	-0.255	-0.547, 0.036	0.09
DBP (mmHg)	-0.238	-0.422, -0.054	0.01	-0.218	-0.410, -0.026	0.03

	OR	95% CI	P	OR	95% CI	P
WtHR						
Healthy	Ref.			Ref.		
High	0.87	0.80, 0.96	0.004	0.90	0.82, 1.00	0.04
BMIz						
Healthy	Ref.			Ref.		
Overweight	0.95	0.88, 1.03	0.25	0.97	0.89, 1.06	0.47
NWO						
No	Ref.			Ref.		
Yes	0.91	0.85, 0.98	0.01	0.97	0.90, 1.04	0.42

β: beta regression coefficient, CI: confidence interval, P: p-value, BMIz: body mass index z-score, Log: logarithmic transformation, WtHR: waist to height ratio, WC: waist circumference, FM: fat mass, SBP: systolic blood pressure, DBP: diastolic blood pressure, OR: odds ratio, NWO: normal weight obesity

Note: WtHR: Healthy ≤0.49, High ≥0.50; BMIz: Healthy ≤1.33 standard deviation (SD), Overweight ≥1.34 SD.

Model 3 adjusted for child's age, child's sex, pubertal status, mother's age, mother's BMI, mother's education level and household social class. Model 4 additionally adjusted for diet quality scores and energy intake.

Table S8. Sensitivity analysis with complete (3 days) dietary data. Longitudinal associations between eating window (hours) at age 7 and outcomes at age 24

	Model 2			Model 3		
	β	95% CI	P	β	95% CI	P
Log BMI	0.004	-0.004, 0.011	0.36	-0.001	-0.009, 0.007	0.82
Log WtHR	0.001	-0.005, 0.007	0.74	-0.000	-0.007, 0.006	0.88
Log WC (cm)	0.001	-0.005, 0.007	0.74	-0.002	-0.008, 0.004	0.52
FM (%)	-0.029	-0.367, 0.309	0.87	-0.130	-0.474, 0.214	0.46
SBP (mmHg)	-0.034	-0.512, 0.444	0.89	-0.273	-0.761, 0.214	0.27
DBP (mmHg)	0.080	-0.291, 0.450	0.67	0.005	-0.375, 0.385	0.98
TC (mmol/L)	-0.012	-0.054, 0.030	0.57	-0.008	-0.051, 0.035	0.72
HDL-C (mmol/L)	-0.003	-0.023, 0.017	0.75	-0.003	-0.023, 0.018	0.80
LDL-C (mmol/L)	0.001	-0.037, 0.038	0.97	0.003	-0.035, 0.042	0.86
Log triglycerides	-0.016	-0.037, 0.005	0.13	-0.014	-0.036, 0.007	0.19
FG (mmol/L)	0.000	-0.031, 0.031	0.99	0.006	-0.026, 0.037	0.72

	OR	95% CI	P	OR	95% CI	P
WtHR						
Healthy	Ref.			Ref.		
High	0.99	0.89, 1.11	0.89	0.97	0.86, 1.08	0.57
BMI						
Healthy	Ref.			Ref.		
High	1.00	0.90, 1.11	0.98	0.95	0.86, 1.06	0.37
NWO						
No	Ref.			Ref.		
Yes	1.04	0.94, 1.15	0.50	1.06	0.95, 1.17	0.30

β: beta regression coefficient, CI: confidence interval, P: p-value, Log: logarithmic transformation, BMI: body mass index, WtHR: waist to height ratio, WC: waist circumference, FM: fat mass, SBP: systolic blood pressure, DBP: diastolic blood pressure, TC: total cholesterol, HDL-C: high density lipoprotein cholesterol, LDL-C: low density lipoprotein cholesterol, FG: fasting glucose, OR: odds ratio, NWO: normal weight obesity.

Note: WtHR: Healthy ≤0.49, High ≥0.50; BMI: Healthy ≤24.9 kg/m^2, Overweight ≥25 kg/m^2.

Model 2 adjusted for for child's age, child's sex, mother's age, mother's BMI, mother's education level and household social class. Model 3 additionally adjusted for diet quality scores and energy intake.

Table S9. Sensitivity analysis with complete (3 days) dietary data. Longitudinal associations between eating window (hours) at age 13 and outcomes at age 24

	Model 2			Model 3		
	β	95% CI	P	β	95% CI	P
Log BMI	-0.005	-0.011, 0.002	0.16	-0.004	-0.010, 0.003	0.28
Log WtHR	-0.002	-0.007, 0.003	0.38	-0.001	-0.006, 0.004	0.77
Log WC	-0.001	-0.005, 0.004	0.84	-0.000	-0.005, 0.005	0.95
FM (%)	-0.384	-0.650, -0.118	0.005	-0.236	-0.512, 0.041	0.09
SBP (mmHg)	0.296	-0.070, 0.663	0.11	0.306	-0.075, 0.688	0.12
DBP (mmHg)	0.073	-0.221, 0.367	0.63	0.185	-0.122, 0.491	0.24
TC (mmol/L)	-0.021	-0.055, 0.013	0.22	-0.018	-0.053, 0.017	0.32
HDL-C (mmol/L)	-0.011	-0.027, 0.005	0.19	-0.015	-0.032, 0.002	0.09
LDL-C (mmol/L)	-0.003	-0.033, 0.027	0.84	0.004	-0.028, 0.035	0.82
Log triglycerides	-0.010	-0.027, 0.008	0.28	-0.008	-0.026, 0.010	0.38
FG (mmol/L)	-0.001	-0.024, 0.021	0.90	-0.003	-0.027, 0.020	0.79

	OR	95% CI	P	OR	95% CI	P
WtHR						
Healthy						
High	0.98	0.89, 1.07	0.59	1.00	0.91, 1.10	1.00
BMI						
Healthy						
High	0.99	0.92, 1.08	0.90	1.01	0.93, 1.10	0.78
NWO						
No						
Yes	0.91	0.84, 0.99	0.03	0.93	0.85, 1.01	0.09

β: beta regression coefficient, CI: confidence interval, P: p-value, Log: logarithmic transformation, BMI: body mass index, WtHR: waist to height ratio, WC: waist circumference, FM: fat mass, SBP: systolic blood pressure, DBP: diastolic blood pressure, TC: total cholesterol, HDL-C: high density lipoprotein cholesterol, LDL-C: low density lipoprotein cholesterol, FG: fasting glucose, OR: odds ratio, NWO: normal weight obesity.

Note: WtHR: Healthy ≤0.49, High ≥0.50; BMI: Healthy ≤24.9 kg/m^2, Overweight ≥25 kg/m^2.

Model 2 adjusted for for child's age, child's sex, mother's age, mother's BMI, mother's education level and household social class. Model 3 additionally adjusted for diet quality scores and energy intake.

Table S10. Sensitivity analysis with both weekday and weekend dietary data. Associations between eating window (hours) and outcomes at age 7

	Model 2			Model 3		
	β	95% CI	P	β	95% CI	P
BMIz	0.052	0.016, 0.087	0.004	0.023	-0.013, 0.059	0.22
Log WtHR	0.002	-0.000, 0.005	0.09	0.002	-0.001, 0.005	0.13
Log WC	0.003	-0.000, 0.006	0.07	0.000	-0.003, 0.003	0.82
SBP (mmHg)	0.123	-0.201, 0.447	0.46	-0.116	-0.446, 0.215	0.49
DBP (mmHg)	-0.035	-0.272, 0.201	0.77	-0.149	-0.392, 0.093	0.23

	OR	95% CI	P	OR	95% CI	P
WtHR						
Healthy						
High	1.06	0.92, 1.22	0.44	1.10	0.95, 1.27	0.22
BMIz						
Healthy						
High	1.13	1.00, 1.27	0.04	1.10	0.97, 1.24	0.13

β: beta regression coefficient, CI: confidence interval, P: p-value, BMIz: body mass index z-score, Log: logarithmic transformation, WtHR: waist to height ratio, WC: waist circumference, SBP: systolic blood pressure, DBP: diastolic blood pressure, OR: odds ratio.

Note: WtHR: Healthy ≤0.49, High ≥0.50; BMIz: Healthy ≤1.33 standard deviation (SD), Overweight ≥1.34 SD.

Model 2 adjusted for for child's age, child's sex, mother's age, mother's BMI, mother's education level and household social class. Model 3 additionally adjusted for diet quality scores and energy intake.

Table S11. Sensitivity analysis with weekday and weekend dietary data. Associations between eating window (hours) and outcomes at age 13

	Model 3			Model 4		
	β	95% CI	P	β	95% CI	P
BMIz	-0.028	-0.060, 0.003	0.08	-0.021	-0.054, 0.011	0.20
Log WtHR	-0.004	-0.007, -0.001	0.006	-0.003	-0.006, 0.000	0.06
Log WC	-0.004	-0.007, -0.001	0.02	-0.004	-0.007, -0.000	0.02
FM (%)	-0.532	-0.781, -0.284	<0.001	-0.276	-0.533, -0.019	0.04
SBP (mmHg)	0.023	-0.268, 0.314	0.88	-0.115	-0.420, 0.190	0.46
DBP (mmHg)	-0.233	-0.429, -0.038	0.02	-0.212	-0.417, -0.007	0.04
	OR	95% CI	P	OR	95% CI	P
WtHR						
Healthy	Ref.			Ref.		
High	0.90	0.81, 1.00	0.04	0.95	0.85, 1.06	0.35
BMIz						
Healthy	Ref.			Ref.		
Overweight	0.97	0.89, 1.06	0.54	1.01	0.92, 1.11	0.84
NWO						
No	Ref.			Ref.		
Yes	0.90	0.83, 0.97	0.008	0.95	0.88, 1.04	0.26

β: beta regression coefficient, CI: confidence interval, P: p-value, BMIz: body mass index z-score, Log: logarithmic transformation, WtHR: waist to height ratio, WC: waist circumference, FM: fat mass, SBP: systolic blood pressure, DBP: diastolic blood pressure, OR: odds ratio, NWO: normal weight obesity.

Note: WtHR: Healthy ≤0.49, High ≥0.50; BMIz: Healthy ≤1.33 standard deviation (SD), Overweight ≥1.34 SD.

Model 3 adjusted for child's age, child's sex, pubertal status, mother's age, mother's BMI, mother's education level and household social class. Model 4 additionally adjusted for diet quality scores and energy intake.

Table S12. Sensitivity analysis with weekday and weekend dietary data. Longitudinal associations between eating window (hours) at age 7 and outcomes at age 24

	Model 2			Model 3		
	β	95% CI	P	β	95% CI	P
Log BMI	0.002	-0.006, 0.010	0.59	-0.002	-0.010, 0.006	0.69
Log WtHR	-0.001	-0.007, 0.005	0.73	-0.002	-0.008, 0.004	0.51
Log WC	-0.001	-0.007, 0.005	0.82	-0.003	-0.010, 0.003	0.30
FM (%)	-0.156	-0.501, 0.188	0.37	-0.228	-0.580, 0.124	0.20
SBP (mmHg)	-0.103	-0.592, 0.386	0.68	-0.304	-0.804, 0.197	0.23
DBP (mmHg)	-0.137	-0.524, 0.250	0.49	-0.194	-0.592, 0.204	0.34
TC (mmol/L)	-0.029	-0.071, 0.014	0.19	-0.025	-0.069, 0.019	0.27
HDL-C (mmol/L)	-0.003	-0.024, 0.018	0.79	-0.001	-0.023, 0.020	0.91
LDL-C (mmol/L)	-0.015	-0.053, 0.024	0.45	-0.015	-0.054, 0.025	0.47
Log triglycerides	-0.020	-0.042, 0.001	0.07	-0.019	-0.041, 0.003	0.09
FG (mmol/L)	0.002	-0.030, 0.034	0.90	0.008	-0.025, 0.040	0.65

	OR	95% CI	P	OR	95% CI	P
WtHR						
Healthy	Ref.			Ref.		
High	1.00	0.89, 1.12	0.94	0.98	0.87, 1.10	0.76
BMI						
Healthy	Ref.			Ref.		
Overweight	0.99	0.89, 1.10	0.82	0.95	0.86, 1.06	0.39
NWO						
No	Ref.			Ref.		
Yes	0.99	0.89, 1.10	0.87	1.01	0.91, 1.12	0.83

β: beta regression coefficient, CI: confidence interval, P: p-value, Log: logarithmic transformation, BMI: body mass index, WtHR: waist to height ratio, WC: waist circumference, FM: fat mass, SBP: systolic blood pressure, DBP: diastolic blood pressure, TC: total cholesterol, HDL-C: high density lipoprotein cholesterol, LDL-C: low density lipoprotein cholesterol, FG: fasting glucose, OR: odds ratio, NWO: normal weight obesity.

Note: WtHR: Healthy ≤0.49, High ≥0.50; BMI: Healthy ≤24.9 kg/m^2, Overweight ≥25 kg/m^2.

Model 2 adjusted for for child's age, child's sex, mother's age, mother's BMI, mother's education level and household social class. Model 3 additionally adjusted for diet quality scores and energy intake.

Table S13. Sensitivity analysis with weekday and weekend dietary data. Longitudinal associations between eating window (hours) at age 13 and outcomes at age 24

	Model 2			Model 3		
	β	95% CI	P	β	95% CI	P
Log BMI	-0.005	-0.012, 0.001	0.11	-0.004	-0.011, 0.003	0.23
Log WtHR	-0.002	-0.007, 0.003	0.35	-0.001	-0.006, 0.005	0.80
Log WC	-0.001	-0.006, 0.004	0.63	-0.000	-0.006, 0.005	0.90
FM (%)	-0.298	-0.574, -0.022	0.03	-0.118	-0.405, 0.169	0.42
SBP (mmHg)	0.216	-0.165, 0.597	0.27	0.254	-0.145, 0.654	0.21
DBP (mmHg)	0.164	-0.142, 0.470	0.29	0.311	-0.009, 0.631	0.06
TC (mmol/L)	0.004	-0.031, 0.039	0.82	0.012	-0.025, 0.049	0.54
HDL-C (mmol/L)	0.011	-0.006, 0.029	0.20	0.009	-0.010, 0.027	0.36
LDL-C (mmol/L)	0.001	-0.031, 0.033	0.93	0.010	-0.023, 0.044	0.55
Log triglycerides	-0.011	-0.029, 0.007	0.23	-0.008	-0.027, 0.011	0.43
FG (mmol/L)	0.003	-0.021, 0.027	0.80	0.002	-0.023, 0.027	0.86

	OR	95% CI	P	OR	95% CI	P
WtHR						
Healthy	Ref.			Ref.		
High	0.97	0.88, 1.06	0.51	0.99	0.90, 1.09	0.80
BMI						
Healthy	Ref.			Ref.		
Overweight	0.98	0.90, 1.07	0.72	0.99	0.91, 1.09	0.90
NWO						
No	Ref.			Ref.		
Yes	0.96	0.89, 1.05	0.40	0.99	0.90, 1.08	0.81

β: beta regression coefficient, CI: confidence interval, P: p-value, Log: logarithmic transformation, BMI: body mass index, WtHR: waist to height ratio, WC: waist circumference, FM: fat mass, SBP: systolic blood pressure, DBP: diastolic blood pressure, TC: total cholesterol, HDL-C: high density lipoprotein cholesterol, LDL-C: low density lipoprotein cholesterol, FG: fasting glucose, OR: odds ratio, NWO: normal weight obesity.

Note: WtHR: Healthy ≤0.49, High ≥0.50; BMI: Healthy ≤24.9 kg/m^2, Overweight ≥25 kg/m^2.

Model 2 adjusted for for child's age, child's sex, mother's age, mother's BMI, mother's education level and household social class. Model 3 additionally adjusted for diet quality scores and energy intake.
